# Temporal dynamics of adenovirus 5 gene expression in normal human cells

**DOI:** 10.1371/journal.pone.0211192

**Published:** 2019-01-24

**Authors:** Leandro Crisostomo, Andrea Michelle Soriano, Megan Mendez, Drayson Graves, Peter Pelka

**Affiliations:** 1 Department of Microbiology, University of Manitoba, Winnipeg, Manitoba, Canada; 2 Department of Medical Microbiology, University of Manitoba, Winnipeg, Manitoba, Canada; University of St Andrews, UNITED KINGDOM

## Abstract

Adenovirus executes a finely tuned transcriptional program upon infection of a cell. To better understand the temporal dynamics of the viral transcriptional program we performed highly sensitive digital PCR on samples extracted from arrested human lung fibroblasts infected with human adenovirus 5 strain *dl*309. We show that the first transcript made from viral genomes is the virus associated non-coding RNA, in particular we detected abundant levels of virus associated RNA II four hours after infection. Activation of E1 and E4 occurred nearly simultaneously later in infection, followed by other early genes as well as late genes. Our study determined that genomes begin to replicate between 29 and 30 hours after infection. This study provides a comprehensive view of viral mRNA steady-state kinetics in arrested human cells using digital PCR.

## Introduction

Adenoviruses are a family of small DNA viruses that infect a variety of human and vertebrate tissues and cell types [1]. Due to the state of the infected cell that is generally restrictive to viral replication, evolution has shaped adenoviral proteins into exquisite reprogrammers of the cell to ensure productive viral replication [1]. Successful viral replication requires that the viral proteins are expressed in an orderly fashion. This enables the reprogramming of the infected cell without alerting the immune system to the infection, or leading to the cell instituting an antiviral defensive program that would lead to abortive infection. The adenovirus capsid is relatively small and does not allow for packaging of an extensive cohort of viral proteins to kick-start the replicative program [1]; rather, the virus packs a very limited number of proteins that are mostly associated with the viral genome and all proteins that carry out cellular reprogramming are expressed *de novo* after the viral genome is delivered to the host cell nucleus [1]. During human adenovirus (HAdV) infection, the first gene expressed is *E1A* from a promoter located at the left end of the genome [1]. Expression of *E1A* is thought to be governed mainly by cellular factors [2], although E1A is able interact with *E1A*-promoter bound factors and enhance its own expression [3, 4]. Upon E1A expression, other early proteins are expressed that are generally thought to be driven by E1A in coordination with the cellular transcriptional machinery, and includes the E2, E3 and E4 transcriptional units [3, 4]. Early in the infection, the Major Late Promoter (MLP) expresses only a small subset of mRNAs and is largely supressed [5]. Consequently, expression of late proteins is low in the early phases of replication; however, this promoter becomes activated with commencement of the viral genome replication leading to high levels of late protein expression and accumulation of all late transcripts [5, 6]. Expression of late proteins is also regulated by products of the L4 gene, which activates late protein expression after initiation of viral genome replication [7]. E1A plays a role in the regulation of expression for all early transcriptional units as well as the MLP as it has been shown to be associated with all of these promoters [8]. In addition, viral non-coding RNAs are expressed early in infection utilizing the cellular RNA Polymerase III while all other viral transcription is performed by the cellular RNA Polymerase II [9]. Overall, the virus carries out a highly orchestrated transcriptional program that converts a non-permissive cell into a permissive one in an efficient manner and within hours of infection.

There are no recent studies of viral transcriptional kinetics that have used modern and highly sensitive techniques for transcript identification and quantification. Prior studies conducted in the 1980s have used relatively insensitive methods of identifying transcripts using hybridization-based techniques, such as those done by Glenn and Ricciardi [10] or Binger and Flint [11]. Although these techniques were state-of-the-art at the time, there is a gap in knowledge using modern techniques that are much more sensitive. Furthermore, early studies were carried out in transformed cell lines limiting the utility of these results to those systems but also, importantly, lacking a comparison to normal cells. More recent studies have focused largely on global transcriptional analysis at fixed time-points with a focus on cellular transcripts, or time points that are widely spaced out giving a limited window into the viral transcriptional program [12–15]. Although some of these studies have looked at kinetics of viral gene expression or gene replication, they looked at a limited subset of genes giving a narrow picture of the viral transcriptional program. A thorough understanding of viral mRNA kinetics is important not only for the understanding of the viral replicative cycle but also for the design and development of viral vectors and oncolytic viruses for therapy.

In the present study, we employed the highly sensitive digital PCR system [16] to examine expression of viral early and late transcripts during the first 36 hours of infection of cell cycle arrested lung fibroblasts IMR-90 with HAdV5 mutant *dl*309. We have chosen this mutant virus as it is the backbone of many viral vectors, and many mutant viruses are derived from this mutant. Our results show that the first viral transcripts arise from the non-coding Virus Associated (VA) RNA, which was evident by an increase in VAII RNA level at approximately 3–4 hours after infection. We also examined expression of viral early and late transcripts in the first 36 hours of infection. Together, our results define a timeline of viral mRNA accumulation in arrested human lung fibroblasts during early phases of viral replicative cycle.

## Materials and methods

### Cell and virus culture

IMR-90 (ATCC# CCL-186) cells were grown in Dulbecco’s Modified Eagle’s Medium (HyClone) supplemented with 10% fetal bovine serum (Seradigm), streptomycin and penicillin (HyClone) at manufacturer-specified concentrations. All virus infections were carried out in serum-free media for 1 hour after which saved complete media was added without removal of the infection media. Cell cycle arrest was achieved by growing the cells to confluence and allowing them to arrest for 72 hours prior to infection, as we have done previously [17, 18].

### PCR primers and probes

Primers and probes used are listed below (F—forward, P—probe, R—reverse). All probes were synthesized by Integrated DNA Technologies and fluorophore used was FAM with two quenchers ZEN and Iowa Black FQ.

**E1A:**
F: tgtgtctgaacctgagcctg
P: agcccgagccagaaccggagcctgcaa
R: atagcaggcgccattttagg

**E1B-19k:**
F: ttctgctgtgcgtaacttgc
P: tggaacagagctctaacagtacctcttggttttggag
R: actaactttgcctgggatgagc

**E1B-55K:**
F: aactgtcacctgctgaagacc
P: acgtagccagccactctcgcaaggcctgg
R: acccaaatgcaaggaacagc

**DNA POL:**
F: accatggtgtcatagtccagc
P: agcttgcccttggaggaggcgccgca
R: agctctacgccctcaaaagtc

**52K encapsidation protein (EP):**
F: atcctgcagagcatagtggtg
P: agcctggctgacaaggtggccgcca
R: tcttgcgggcgtaaaacttg

**pIIIa:**
F: tccatggttgcactaaacgc
P: agtacacagcccgccaacgtgccgcg
R: cgctcacaaagttggtgtagtc

**III (Penton Base, Capsid Protein III):**
F: agcaatgacagcaccttcac
P: acggcgaccctcagaccggaatccgct
R: ttacgtcaggagtgcaaagc

**Hexon:**
F: agcatttgcctttacgccac
P: tggcccacaacaccgcctccacgctt
R: tgtcgtttctaagcatggcc

**DNA Binding Protein:**
F: tttcttcttgggcgcaatgg
P: aggtcgatggccgcgggctgggtgt
R: aagactcatcacaagacgcg

**100K (L4 Hexon Assembly Protein):**
F: tctgcaacaggaaaacagcg
P: agggtgacaacgcgcgcctagccgt
R: acctcgatgctgcgttttag

**E3:**
F: ttgttgccatctctgtgctg
P: acgccaccgtcttcacccgcccaagcaa
R: aaagtaccaggtaaggttcgcc

**Fiber (L5):**
F: atgcttgcgctcaaaatggg
P: tctggacgaggccggcaaccttacctccca
R: tttttgagaggtgggctcac

**E4 orf6/7:**
F: aagttcatgtcgctgtccag
P: agccacaggctgctgtccaacttgcg
R: gtggacttctccttcgccg

**E4 orf3:**
F: gcacaatggcttccaaaagg
P: acggccctcacgtccaagtggacg
R: tttgggcatggttgaaggtg

**VAII RNA:**
F: ttttccaagggttgagtcgc
P: tcgagtctcggaccggccggac
R: aatttgcaagcggggtcttg

Real-time PCR primers for *glyceraldehyde 3-phosphate dehydrogenase* (*GAPDH*) and *E4orf3* were previously described [8, 17–20].

### Gene expression analysis

IMR-90 cells were infected with *dl*309 [21] at an MOI of 50. Total RNA was extracted using the Nucleozol at the indicated time points according to the manufacturer’s instructions. 1 μg of total RNA was used in reverse-transcriptase reaction using SuperScript VILO reverse transcriptase (Invitrogen) according to the manufacturer’s guidelines using random hexanucleotides for priming. The cDNA was subsequently used either for digital quantification using the BioRad QX200 digital droplet PCR system with automatic droplet generator with ddPCR Probe Master Mix, or using SsoAdvanced Master Mix for probes for conventional qRT-PCR analysis on BioRad CFX96 for time points after 17 hours. Analysis of expression data was represented as percentage of *GAPDH* mRNA levels.

### Statistical analysis

Statistics were performed as described by [22]. Briefly, statistical analysis was conducted using one-way analysis of variance (ANOVA) followed by post hoc comparison using Tukey test of viral genes from *dl*309 infection *versus* mock-infected cells. *P*-values were two-tailed and values of < 0.05 were considered statistically significant in gene expression and genome quantification assays.

### Western blot

Cell lysates were prepared by lysing 425,000 of IMR-90 cells in NP-40 lysis buffer, boiling with sample buffer, and resolving on 4–12% Novex Bolt gradient gel, transferring to a PVDF membrane using Genscript’s L1 eBlot and blotting for the specified antibodies. Each lane represents loading of protein from approximately 64,000 cells. E1A was detected using M2, M37, M58, and M73 mouse monoclonal antibodies that were previously described [23] and were used at 1:10 dilution from the hybridoma supernatant. Mouse monoclonal anti-72k E2 DNA Binding Protein (DBP) antibody was previously described [24] and was used at a dilution of 1:400. Viral structural and late proteins were detected with anti-adenovirus type 5 antibody from Abcam (cat # ab6982) at a dilution of 1:5000.

### Viral genome quantification

Infected cells were lysed in lysis buffer (50mM Tris pH 8.1, 10mM EDTA and 1% SDS) on ice for 10 minutes. Lysates were sonicated in a Covaris M220 focused ultrasonicator using the default 500bp fragmentation program in a Covaris microTUBE-500 AFA fiber screw-cap glass tubes to break-up cellular chromatin and subjected to digestion using Proteinase K (NEB) according to manufacturer’s directions. Following digestion viral DNA was purified using EZ-10 Gel Extraction Kit (Bio Basic). PCR reactions were carried out using EvaGreen for QX200 master mix according to manufacturer’s directions using a QX200 digital droplet PCR instrument (BioRad). The primers used were the same as those used for expression analysis of *E4orf3* region, the annealing temperature used was 60°C and 40 cycles were run.

### Viruses

Viruses used in the study were HAdV5 mutant *dl*309 [21] expressing wild-type (wt) E1A but deleted for E3 14.7K, 14.5K, and 10.4K genes [25], and was generously donated by Joe Mymryk. The virus was amplified in low passage 293 cells and were also titered by plaquing on these 293 cells prior to performing assays. All infections were carried out in serum-free medium for 1 hour at a MOI of 50 that ensures that most cells were infected. The MOI chosen was based on infection of arrested IMR-90 cells and immunostaining for E1A expression 24 hours later.

## Results

### Expression of viral early genes in arrested lung fibroblasts

**Fig 1:**
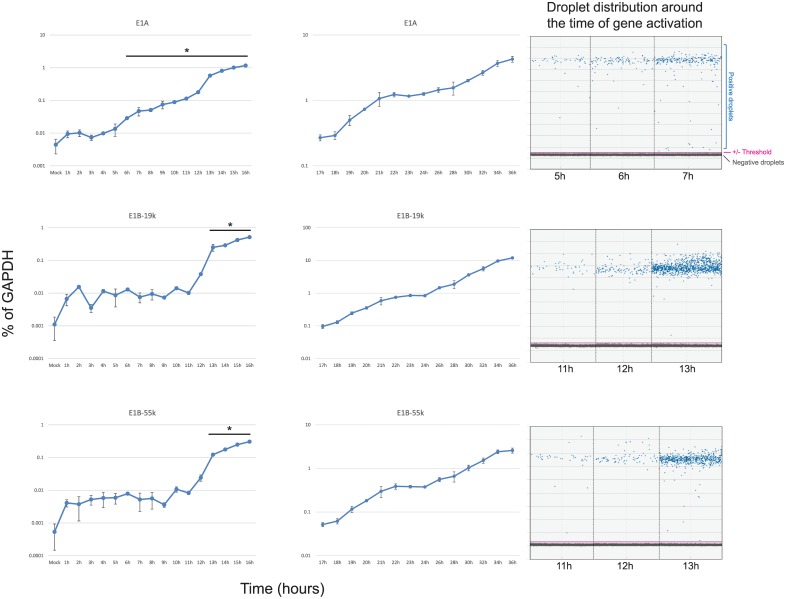
Expression of *E1* during the first 36 hours after infection with HAdV5. Arrested IMR-90 cells were infected with *dl*309 at a MOI of 50 and total RNA was extracted at the indicated time points using Nucleozol reagent, treated with DNase I and reverse-transcribed using VILO. Transcript levels of the indicated viral mRNAs were quantified using the BioRad QX200 droplet digital PCR system for the first 16 hours, and using BioRad CFX96 real-time PCR instrument for 17 to 36 hours, and plotted as percentage of GAPDH expression. Right column shows droplet distribution at the time when transcripts increase above background levels. Error bars represent standard deviation of biological replicates, n = 3. Bar with asterisk represents changes above mock level that are statistically significant with the following *p* values: E1A ≤ 0.001; E1B-19k < 0.0001; E1B-55k < 0.0001. All time-points between 17 and 36 hours were statistically significant.

**Fig 2:**
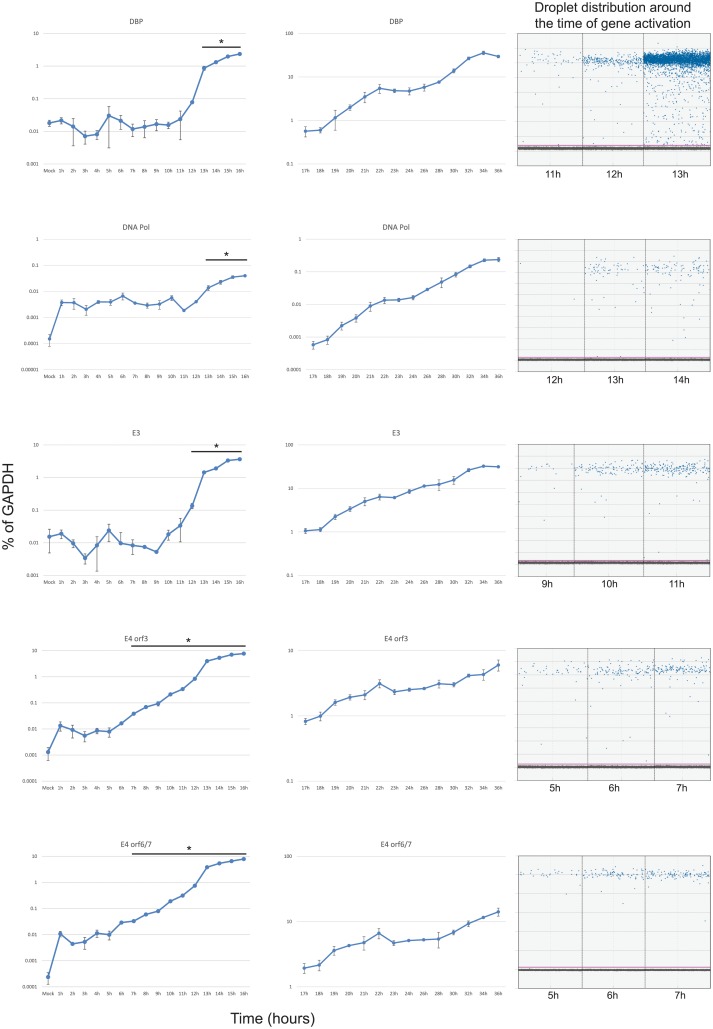
Expression of *E2*, *E3*, and *E4* during the first 36 hours after infection with HAdV5. Arrested IMR-90 cells were infected with *dl*309 at an MOI of 50 and total RNA was extracted at the indicated time points using Nucleozol reagent, treated with DNase I and reverse-transcribed using VILO. Transcript levels of the indicated viral mRNAs were quantified using the BioRad QX200 droplet digital PCR system for the first 16 hours, and using BioRad CFX96 real-time PCR instrument for 17 to 36 hours, and plotted as percentage of GAPDH expression. Right column shows droplet distribution at the time when transcripts increase above background levels. Error bars represent standard deviation of biological replicates, n = 3. Bar with asterisk represents changes above mock level that are statistically significant with the following *p* values: DBP < 0.001; DNA pol ≤ 0.0058; E3 ≤ 0.0086; E4 orf3 < 0.0001; E4 orf6/7 < 0.0001. All time-points between 17 and 36 hours were statistically significant.

**Fig 3:**
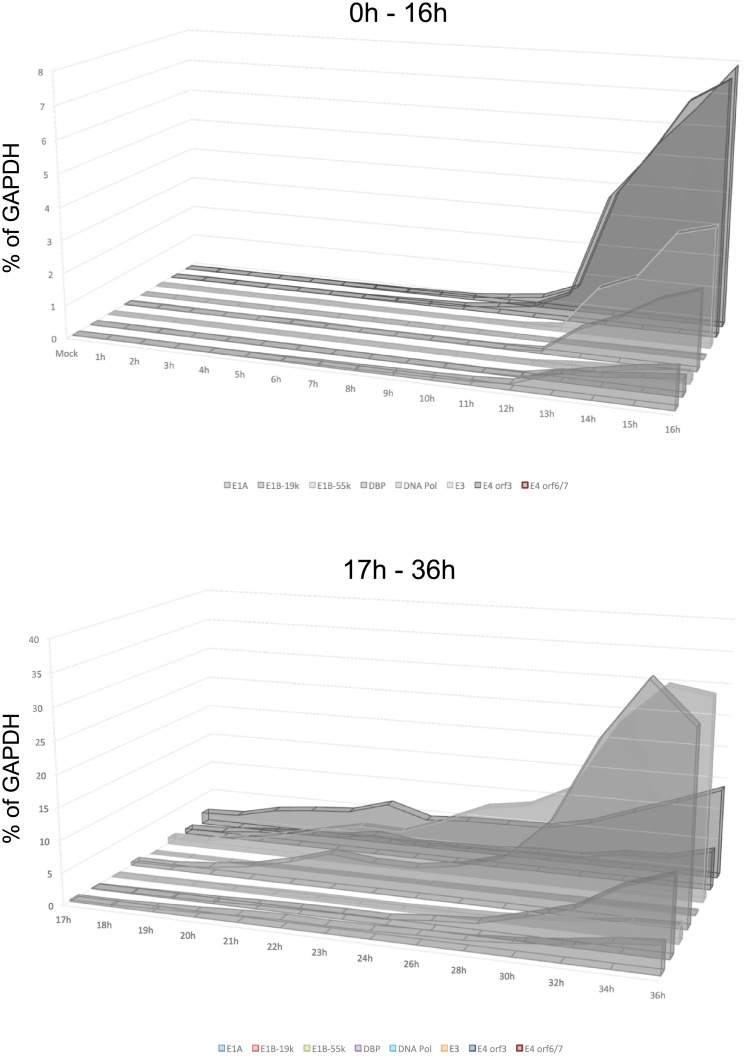
Aggregate results of viral early gene expression. Combined results from Figs 1 and 2 plotted on a linear scale showing relative levels of each viral transcript at the given time point.

To determine the temporal expression pattern of HAdV5 early genes, contact inhibited IMR-90 cells were infected with HAdV5 *dl*309 at a MOI of 50. This MOI ensured that most of the cells were infected with the virus without overwhelming the cells with a high initial viral load and allows for direct comparison with other studies that used the same MOI [10]. Expression of all genes was normalized to cellular GAPDH and was represented as percentage of the GAPDH transcript. We have previously observed that GAPDH mRNA concentration remains steady in IMR-90 cells infected at a similar MOI for up to 48 hours after infection, therefore GAPDH mRNA was an appropriate reference gene [26]. Initially, the levels of E1A mRNA fluctuated around 0.01% of GAPDH levels, with an increase in mRNA levels occurring at the 6 hour mark (Fig 1). E1A transcript levels continued a slow but steady rise until 13 hours when expression started to increase more rapidly. A similar pattern was observed for *E4* genes (Fig 2). Detectable expression of *E3* started at approximately 10 hours. Interestingly, expression from the viral E2 promoter showed only a minor difference between the E2-DNA Binding Protein (E2 DBP) and the E2-Polymerase (E2 Pol), with the former starting to show expression increasing between 11 and 12 hours and the latter at 12 and 13 hours after infection (Fig 2). Expression of E1B transcripts was later than anticipated (Fig 1), with E1B-19k and E1B-55k mRNAs showing little to no expression until 12–13 hours after infection. For most viral early genes, we observed a continuous increase in expression thorough-out the course of the infection (such as *E1A* and *E4*), whereas other early genes seemed to show a levelling off late in the infection (including *E2* and *E3*). We also observed a significant difference in mRNA levels of the E2 transcripts, with the mRNA encoding the E2 Pol being considerably less abundant than the mRNA encoding E2 DBP. It should be noted that time points 17 hours and later were assayed using conventional quantitative real-time PCR (using the same primers and probes as used for digital PCR) and not digital PCR due to much higher concentrations of target sequences in samples, which are unsuitable for digital PCR. It should be noted that the difference in expression levels observed between the results obtained using conventional qPCR and ddPCR were caused by the technique used and their inherent differences, rather than by any actual changes in expression. We have compared overlapping time points between the two different techniques to verify this (data not shown). Aggregate comparison of all early gene expression is shown in Fig 3.

### Expression of viral late genes

**Fig 4:**
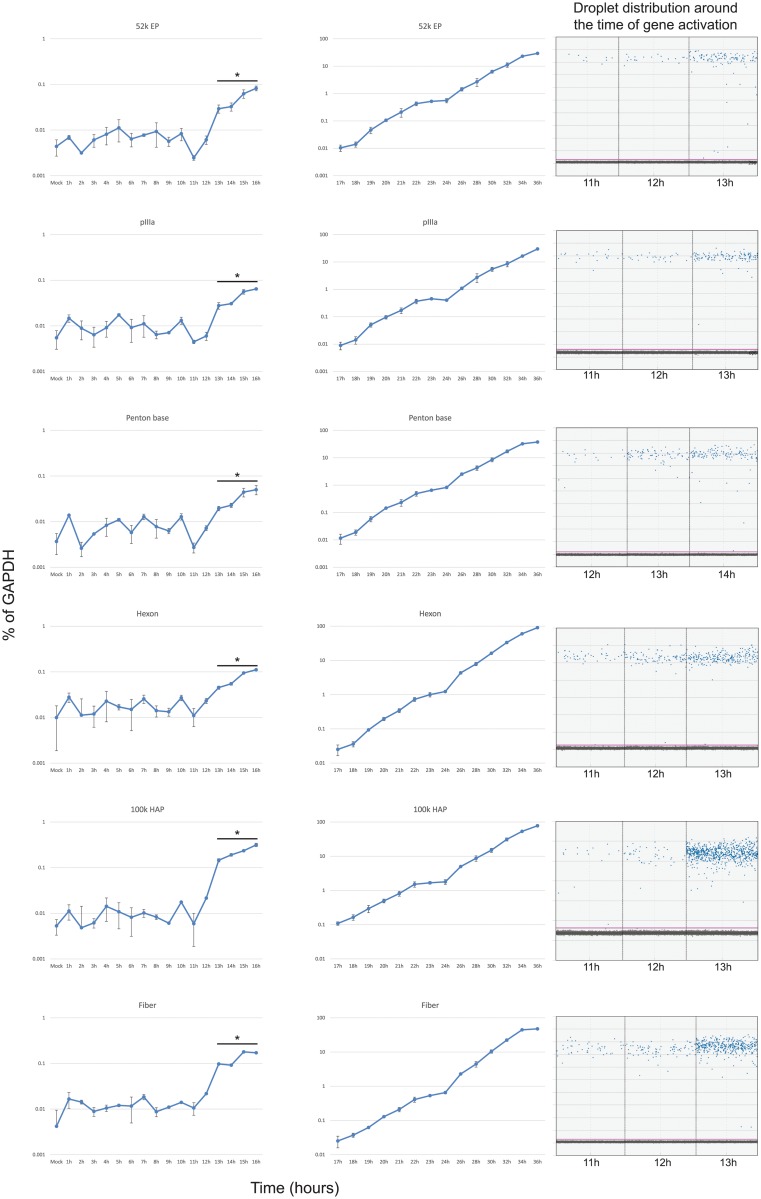
Expression of viral late genes during the first 36 hours after infection with HAdV5. Arrested IMR-90 cells were infected with *dl*309 at a MOI of 50 and total RNA was extracted at the indicated time points using Nucleozol reagent, treated with DNase I and reverse-transcribed using VILO. Transcript levels of the indicated viral mRNAs were quantified using the BioRad QX200 droplet digital PCR system for the first 16 hours, and using BioRad CFX96 real-time PCR instrument for 17 to 36 hours, and plotted as percentage of GAPDH expression. Right column shows droplet distribution at the time when transcripts increase above background levels. Error bars represent standard deviation of biological replicates, n = 3. Bar with asterisk represents changes above mock level that are statistically significant with the following *p* values: 52k EP ≤ 0.001; pIIIa ≤ 0.0006; Penton base ≤ 0.0001; Hexon ≤ 0.0002; 100k HAP ≤ 0.0007; Fiber ≤ 0.0004. All time-points between 17 and 36 hours were statistically significant.

**Fig 5:**
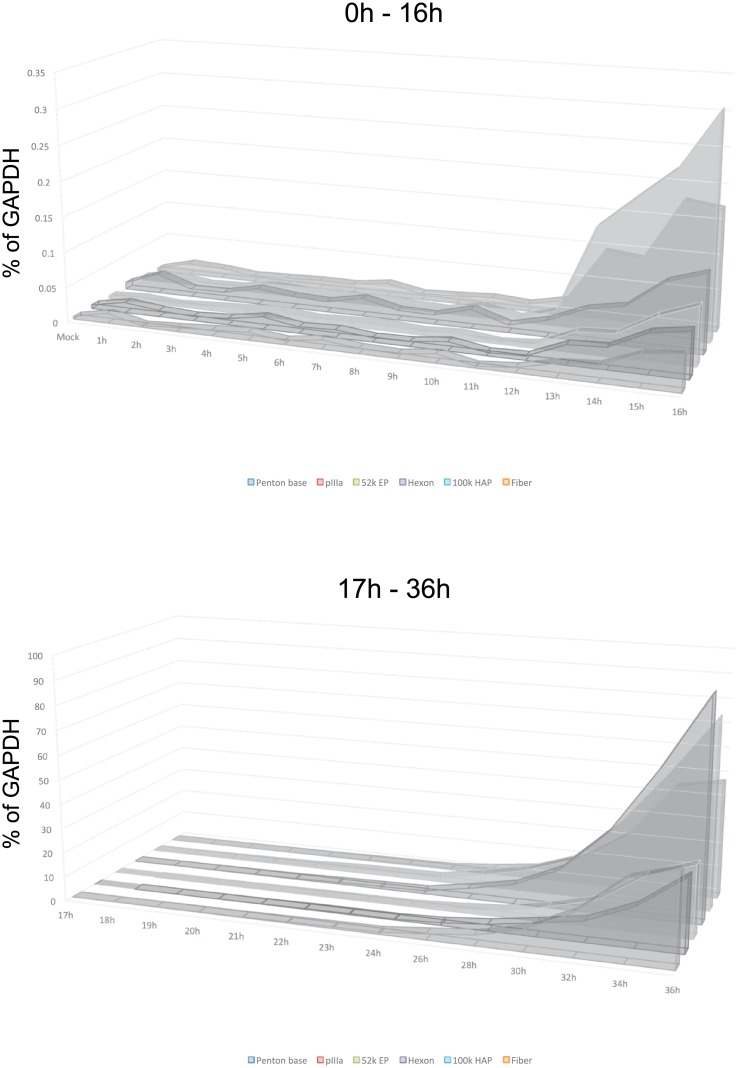
Aggregate results of viral late gene expression. Combined results from Fig 4 plotted on a linear scale showing relative levels of each viral transcript at the given time point.

To assess the temporal dynamics of viral late gene expression we examined representative transcripts from transcriptional units L1 through L5 (52k Encapsidation Protein, 52k EP; pIIIa; Penton base; Hexon; 100k Hexon Assembly Protein; Fiber; Fig 4). Expression of all of these genes started to occur at between 11 and 13 hours after infection and continued a logarithmic increase over the 36-hour length of the assay, with some levelling off observed at the last time point for fiber and penton base mRNAs. Interestingly, we did not observe an upregulation of expression of these late genes previously reported [5] and associated with the initiation of viral genome replication, which we observed to occur at between 29 and 30 hours after infection. Although a slight increase in expression level was observed after 24 hours. Aggregate comparison of all early gene expression is shown in Fig 5.

### Expression of virus associated RNA II

**Fig 6:**
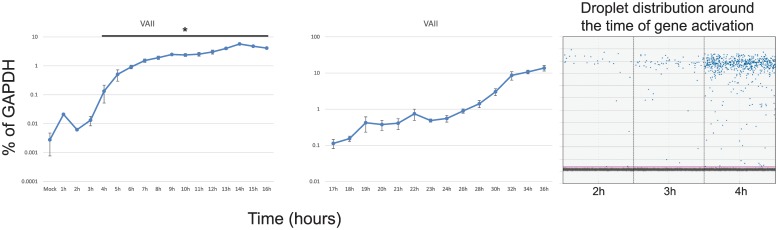
Expression of VAII RNA during the first 36 hours after infection with HAdV5. Arrested IMR-90 cells were infected with *dl*309 at a MOI of 50 and total RNA was extracted at the indicated time points using Nucleozol reagent, treated with DNase I and reverse-transcribed using VILO. Transcript levels of the indicated viral mRNAs were quantified using the BioRad QX200 droplet digital PCR system for the first 16 hours, and using BioRad CFX96 real-time PCR instrument for 17 to 36 hours, and plotted as percentage of GAPDH expression. Right column shows droplet distribution at the time when transcripts increase above background levels. Error bars represent standard deviation of biological replicates, n = 3. Bar with asterisk represents changes above mock level that are statistically significant with the *p* ≤ 0.0027. All time-points between 17 and 36 hours were statistically significant.

HAdV encodes two non-coding RNAs, VA RNA I and II that are expressed early in infection and are involved in suppression of innate antiviral defences [27]. We analyzed expression of VAII RNA after infection (Fig 6) of arrested fibroblasts. We did not examine VAI RNA as all primer/probe triplets that we tested did not give satisfactory results. VAII RNA was expressed earlier than any other gene with the levels of the transcript increasing shortly after infection. Interestingly, the RNA seemed to express in stages, increasing a certain amount before levelling off. This increase occurred around 4 hours, 11 hours, 19 hours, and 24 hours after infection. Overall, the expression of VAII RNA occurred prior to any other gene and continued to increase throughout infection until 36 hours after infection.

### Initiation of viral genome replication

**Fig 7:**
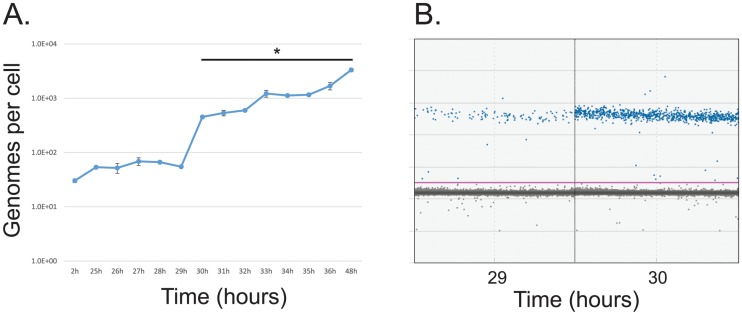
Analysis of viral genome copy number in the first 48 hours of infection with HAdV5. (A) Arrested IMR-90 cells were infected with *dl*309 at a MOI of 50. Viral DNA was extracted at the indicated time points, and absolute quantification of viral genomes using E4orf3 primers was measured using the BioRad QX200 droplet digital PCR system. Viral genomes are plotted on per cell basis. Error bars represent standard deviation of biological replicates, n = 2. Bar with asterisk represents changes above mock level that are statistically significant with *p* ≤ 0.0001. (B) Sample droplet distribution from one of the biological replicates for 29 and 30 hour time points from (A).

To correlate the expression levels of viral genes with viral genome replication we examined viral genome copy numbers after infection (Fig 7). Viral genome levels remained at the input level for the first 29 hours after infection, rapidly increasing at 30 hours after infection and thereafter showing a slow and steady rise. Genome levels were unchanged between 2 and 29 hours after infection, indicative of initial viral input. Together, these results show that at a MOI of 50, in arrested lung fibroblasts IMR-90, genomes begin to replicate between 29 and 30 hours after infection. This is in agreement with our previous results showing viral DNA replication commencing between 24 and 48 hours after infection, and other results showing replication of DNA commencing between 26 and 28 hours in human foreskin fibroblasts at MOI of 30 [15, 17, 18]. These results also suggest that replication of viral genomes is more dependent on the host cell state rather than the expression levels of the viral DNA replication machinery.

### Analysis of viral protein expression

**Fig 8:**
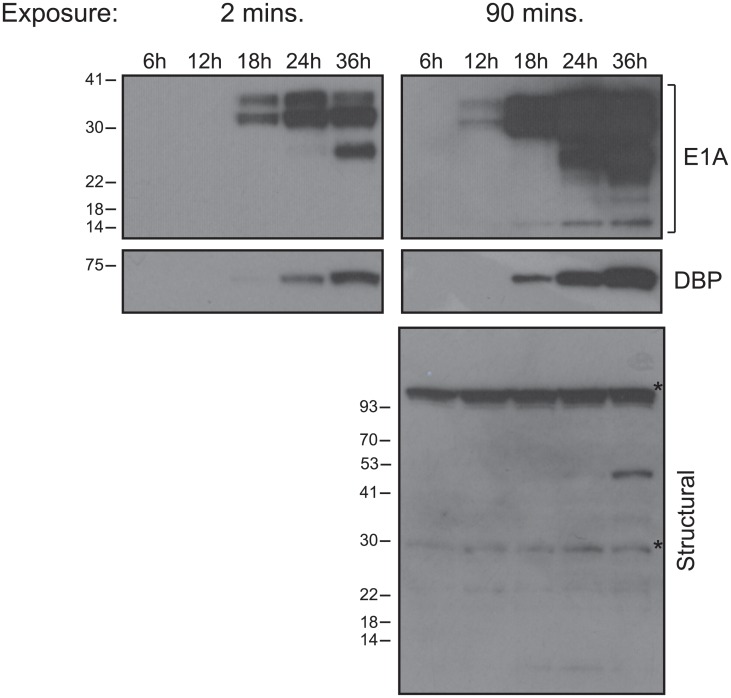
Analysis of viral protein levels over the first 36 hours of infection of arrested IMR-90 cells with HAdV5 *dl*309 at a MOI of 50. Arrested IMR-90 cells were infected at a MOI of 50 for the indicated time period. Cell lysate was prepared by lysing 425,000 cells in NP-40 lysis buffer, boiling with sample buffer, and resolving on 4–12% Novex Bolt gradient gel, followed by western blotting for the indicated proteins. Each lane represents loading of protein from approximately 64,000 cells. Asterisks denote background bands. Relative molecular weights are indicated in kilo-Daltons.

To be able to correlate the steady state mRNA levels with protein levels, we performed western blotting for E1A, DBP, and viral structural proteins at 6, 12, 18, 24, and 36 hours after infection (Fig 8). E1A was detectable at 12 hours after infection, approximately 6 hours after its mRNA levels rose above background (Fig 1). E2 DBP became detectable at 18 hours after infection, also 6 hours after its mRNA rose above background (Fig 2). Interestingly, structural and late proteins were scarcely detectable at 36 hours after infection, despite their transcription rising above background levels at 13 hours after infection.

## Discussion

In the present study we have used the highly sensitive digital PCR combined with probes to evaluate the temporal expression pattern of viral genes early in the infection of arrested IMR-90 cells with HAdV strain *dl*309, since this strain is widely used for generation of other viral mutants and vectors. We have also established the exact time of initiation of viral genome replication and its relation to viral gene expression for this strain and this cell type at a MOI of 50. Lastly, we provide a comparison table (Table 1) with earlier studies performed in HeLa cells. Therefore, our investigation provides a comprehensive picture of viral gene expression that may be of use to those that use the *dl*309 mutant and its derivatives for their studies.

**Table 1:** Comparison of earliest transcript detection times in IMR-90 cells and previously published results from HeLa cells.

Gene	IMR-90^a^	HeLa^b^
**E1A**	6h	1.5—2h
**E1B-19k**	12h	8h
**E1B-55k**	12h	3.5h
**52k EP**	13h	7—8.5h
**E2A**	12—13h	2—3h
**E3**	12h	3h
**E4**	6—7h	3h
**VAII**	4h	~3h

Times given are after the initial application of the virus to the cells and are in hours.

^a^ This study.

^b^ Based on references [10, 11, 30].

One of the first transcripts that was detected using our approach was the Virus Associate RNA II (Fig 6). Although expression of this transcript was detectable above mock sample as early as one hour after infection it is unlikely that this represents *de novo* transcription as we have observed similar uptick for some other early mRNAs. Rather, these observations may reflect peculiarities of the assay at very low target concentrations or incomplete digestion of this small gene with DNase I treatment. Overall, the background levels of all of viral genes was observed to be between 0.005 and 0.01% of GAPDH, with most primer-probe combinations giving less than 5 copies/μL of target sequence amplification in mock samples or samples in the first few hours after infection, establishing a consistent baseline for evaluation of gene activation. Our results show that the viral non-coding RNA is one of the first transcripts expressed, with a ~10-fold increase by 4 hours after infection over mock-infected cells. This is similar to what was previously observed in HeLa cells using hybridization methods [28–30] and shows that non-coding VA RNA is the first transcript expressed after infection. This early activation may stem from the fact that VA RNAs are transcribed by RNA polymerase III, rather than RNA polymerase II as is the case for all other viral genes [9]. It is still unclear how this contributes to the early expression of VA RNA, it is possible that the overall abundance and availability of the polymerase enables early activation.

Our results suggest that expression of viral early genes is somewhat different and occurs later than what was previously reported in infected HeLa cells [10], see comparison Table 1. We observed a modest variance in expression levels in the first few hours of infection (time points 1 to 10 hours after infection). With some mRNAs showing more fluctuations as compared to the mock-infected cells (such as E1B and E3 in Figs 1 and 2). This is likely caused by several factors including the heterogeneity of viral DNA delivery on per-cell basis, as has been previously reported [31], and the overall very low level of signal observed prior to viral gene expression. Nevertheless, we observed similar timing for transcription of the E1A and E4 regions, both rising above background between 5 and 7 hours after infection. Expression of the other early transcripts followed, with E2 DBP and E3 starting to increase at approximately 12 and 10 hours after infection, respectively, further followed by E1B-55k and E1B-19k at 13 hours and 12 hours, respectively. Expression of the E2 Pol was delayed by about 1 hour as compared to E2 DBP. Interestingly, the levels of the polymerase expression were considerably lower than any other early transcript, never crossing 1% level of GAPDH expression. These results are somewhat different from previous hybridization-based techniques carried out in transformed HeLa cells [10], particularly in regards to the timing of onset of transcription of specific genes, but are largely in agreement with more recent studies performed in non-transformed cells [15]. Although, interestingly, a recent study of viral mRNA expression using *in situ* hybridization technique found E1A mRNA levels being detectable relatively late, at 13 hours after infection [12]. It is likely that this difference stems from the method of detection used as well as the lower MOI with which the cells were infected (10 versus 50). Some of our observed differences may also stem from the method of quantification with blot-based techniques as used by [10] and [11] being often more difficult to interpret in particular [32]. Other differences are likely caused by the cell type and cell state. Specifically, we used primary lung fibroblasts that have been cell cycle arrested in G0/G1 by contact inhibition whereas prior studies used cycling HeLa cells already expressing human papilloma virus proteins and which are primed to replicate the viral genome. The two different sets of results also highlight potential differences between adenovirus replication in normal versus transformed cells as previously reported [15], which may be of interest to those developing oncolytic viruses for cancer therapy or viral vectors. It should also be noted that some of the differences may stem from the different virus strains used, as here we used a mutant virus deleted for a portion of the E3 region.

Interestingly, previously in HeLa cells [10], it was observed that levels of some early transcripts would drop in expression after 6 hours. We did not observe this phenomenon, instead we observed some plateauing of expression of E4 transcript in late early phase prior to initiation of genome replication (Fig 2). It is likely that the early techniques used in prior work may have contributed to the observed drop in early gene expression relatively early in infection. It is also possible that some of these difference might be attributed to the cell line or the virus strain used, as we used *dl*309 deletion mutant that is missing a section of the E3 region [21] *versus* wild-type virus used in other studies. The overall expression was somewhat different between E4orf3 and E4orf6/7, particularly later in infection, with overall levels of E4orf6/7 being higher than E4orf3 (Fig 3), which is different than what was previously reported [33] where the opposite was observed. This difference may reflect overall assay sensitivity and contribution of background levels. Since these genes are driven by the same promoter and pre-mRNA is common to all E4 transcripts, this difference likely reflects the overall abundances of the mRNA at a given time. The observed difference, therefore, is most probably caused by differential splicing favouring one transcript versus another.

We observed a steady increase and activation of viral late genes between 11 and 13 hours after infection (Fig 4), which differs from previous reports suggesting that only L1 mRNA encoding the 52k protein is expressed early [5]. Expression of *hexon* continued to rise exponentially throughout the duration of the assay, whereas expression of *fiber* plateaued at around the 32 hour time point after infection. Interestingly, we did not observe activation of the late promoter reported [5] to occur in the late phase following initiation of viral DNA replication, which we observed between 29 and 30 hours after infection. However, we did observe some plateauing of viral late transcripts around 24 hours, followed by an increase in expression at 26 hours onwards. It is possible that this represents the previously reported activation of the late promoter slightly before onset of viral genome replication. Plateauing of the *fiber* mRNA levels was somewhat unexpected and was similar to what we observed for some early genes. Since all late transcripts are driven by the same promoter [6, 34], it is possible that this is driven by splicing and/or RNA stability alterations as is seen for E1A transcripts late in the infection [5, 17, 35]. This likely reflects a greater need for certain structural components, such as hexon protein, shifting production of the more needed mRNA away from those that are less needed due to lower protein demand per capsid. The overall levels of late mRNAs remained relatively low up to 24 hours after infection as compared to some of the early mRNAs. This contradicts early studies that showed that these mRNAs are the most abundant mRNAs at this time of viral replication [36]. This observed difference is likely due to the use of HeLa cells in the study by Flint and Sharp, which will replicate the virus much quicker and allow an earlier entry into the late phase, which is considerably delayed in primary or non-transformed cells [15]. Consistent with this, we did not observe viral genome replication until 30 hours after infection (Fig 7). Therefore, if we look at later time-points, late transcript become much more abundant with hexon mRNA reaching highest levels of expression, in-line with previous reports [36].

Interestingly, we observed a disconnect between structural transcripts and structural protein levels (Figs 4 and 8). Whereas early proteins became detectable by western blot 6 hours after their mRNA rose above background (for example, see E1A mRNA at 6 hours in Fig 1 and E1A protein appearing at 12 hours in Fig 8), some late proteins only (likely either penton/penton base or protein V) became faintly detectable at 36 hours after infection, nearly 24 hours later. This suggests that late mRNAs are subject to translational repression of some kind. Perhaps they are not exported efficiently due to the low levels of E1B-55k at earlier time points in infection, a protein essential for efficient export and translation of viral transcripts driven by the MLP [37]. Nevertheless, this is consistent with previous observations where late proteins were only detectable at 48 hours after infection of arrested lung fibroblasts [17, 18].

In conclusion, our study has examined the transcriptional kinetics of viral and cellular gene expression in arrested lung fibroblasts, IMR-90, infected with HAdV5. We provide a comprehensive view of viral gene expression in the first 36 hours of infection, showing that the first viral transcript expressed is not E1A but, rather, the non-coding RNA VAII. The results we present here also show that other viral transcriptional units are activated nearly simultaneously, particularly E1A and E4. Our results precisely establish the time at which viral genomes begin to replicate. Lastly, we provide new insight into late gene expression, showing that the MLP is active from early phases of infection generating mRNAs encoding all late transcriptional units, but with little protein produced from these mRNAs. These results provide a comprehensive framework of early viral gene expression, with insights into initiation of viral genome replication and its dynamics.
