# Correction: Temporal dynamics of adenovirus 5 gene expression in normal human cells

**DOI:** 10.1371/journal.pone.0213211

**Published:** 2019-02-26

**Authors:** The PLOS ONE Staff

Fig 3 and Fig 5 were published in greyscale instead of color. The publisher apologizes for the error. Please see the correct Fig 3 and Fig 5 here.

**Fig 3:**
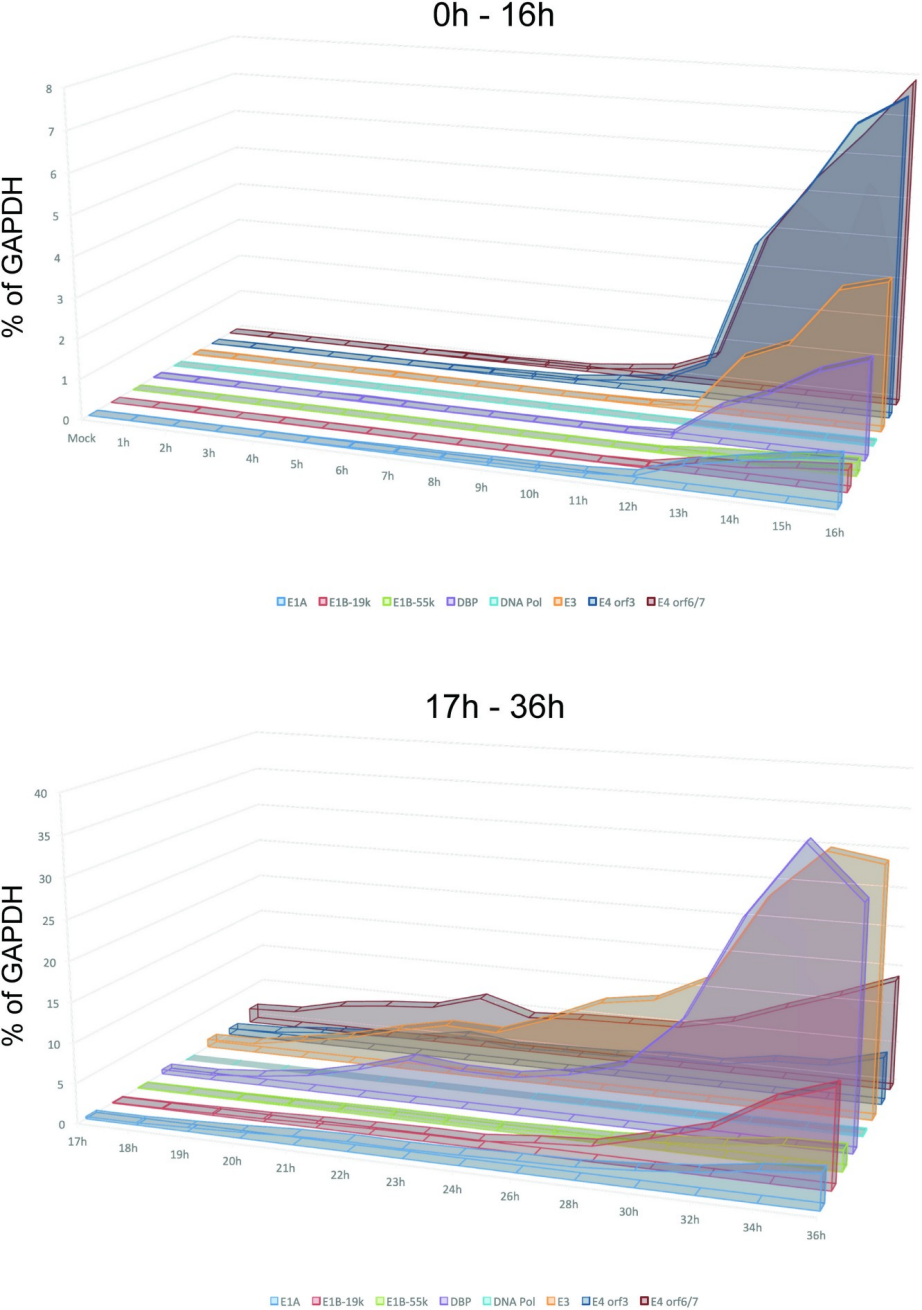
Aggregate results of viral early gene expression. Combined results from Figs 1 and 2 plotted on a linear scale showing relative levels of each viral transcript at the given time point.

**Fig 5:**
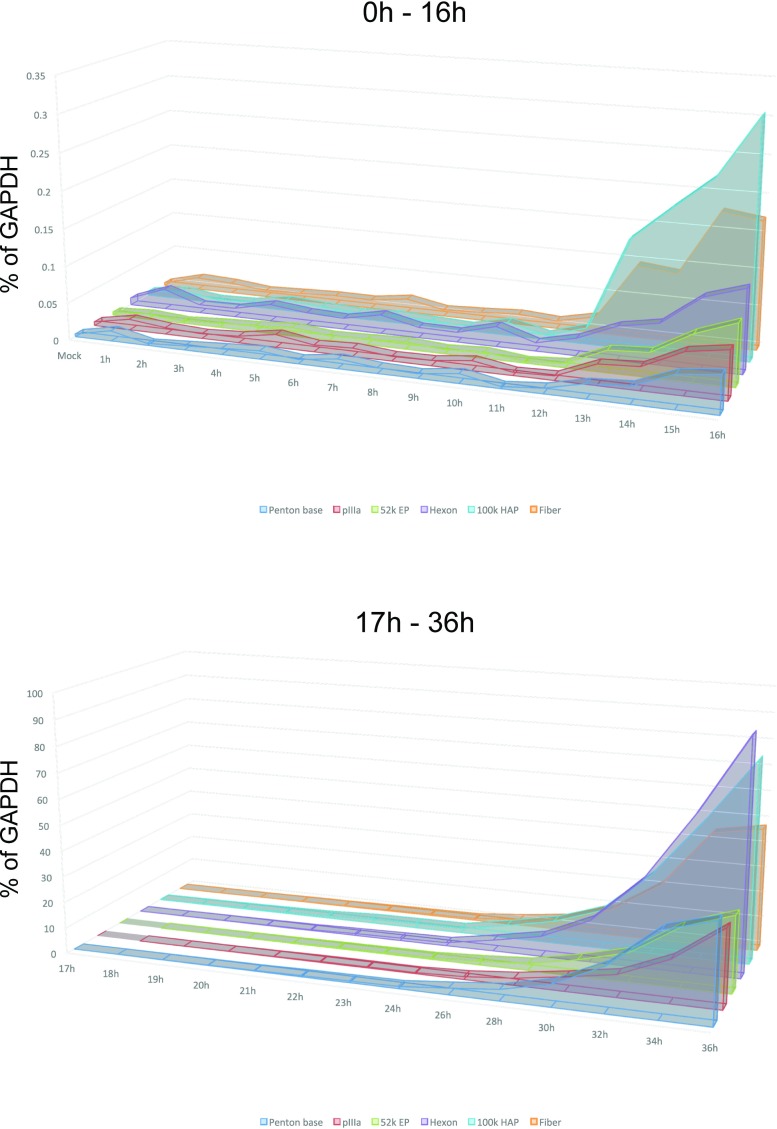
Aggregate results of viral late gene expression. Combined results from Fig 4 plotted on a linear scale showing relative levels of each viral transcript at the given time point.
